# Growth of Laser-Induced Microbubbles inside Capillary Tubes Affected by Gathered Light-Absorbing Particles

**DOI:** 10.3390/mi13050740

**Published:** 2022-05-06

**Authors:** Jia-Wen He, Hao-Dong Wang, Bo-Wei Li, Wen Bai, Dong Chen, Min-Cheng Zhong

**Affiliations:** Anhui Province Key Laboratory of Measuring Theory and Precision Instrument, School of Instrument Science and Opto-Electronics Engineering, Hefei University of Technology, Hefei 230009, China; hejiawen@mail.hfut.edu.cn (J.-W.H.); haodongwang@mail.hfut.edu.cn (H.-D.W.); libowei@mail.hfut.edu.cn (B.-W.L.); baiwen@mail.hfut.edu.cn (W.B.); dchen@hfut.edu.cn (D.C.)

**Keywords:** microbubble, laser heating, bubble growth, capillary tube, Marangoni convection, light-absorbing particle

## Abstract

Microbubbles have important applications in optofluidics. The generation and growth of microbubbles is a complicated process in microfluidic channels. In this paper, we use a laser to irradiate light-absorbing particles to generate microbubbles in capillary tubes and investigate the factors affecting microbubble size. The results show that the key factor is the total area of the light-absorbing particles gathered at the microbubble bottom. The larger the area of the particles at bottom, the larger the size of the microbubbles. Furthermore, the area is related to capillary tube diameter. The larger the diameter of the capillary tube, the more particles gathered at the bottom of the microbubbles. Numerical simulations show that the Marangoni convection is stronger in a capillary tube with a larger diameter, which can gather more particles than that in a capillary tube with a smaller diameter. The calculations show that the particles in contact with the microbubbles will be in a stable position due to the surface tension force.

## 1. Introduction

Microbubbles are generally defined as bubbles with an outer diameter of less than one millimeter but larger than one micrometer [1]. Microbubbles have important applications in many fields [2,3,4,5,6,7,8,9]. One important application is for manipulating the particles in microfluidic channels assisted by manipulating the microbubbles. Microbubbles in microfluidic channels can exist as an interface in liquids and can be used to apply external forces for manipulation by using the different acoustic, optical, and thermodynamic properties between the gas inside the microbubble and the surrounding liquid. The external force applied at the gas–liquid interface can be used to manipulate the microparticles and the microbubbles in a non-contact manner. This non-contact manipulation property allows for unique and effective applications of microbubbles in microfluidics, which have a wide range of applications [1].

External forces are always originated from the flow, which is affected by the boundaries of microfluidic channels. Modulation of the flow field helps to manipulate the bubbles precisely. A capillary tube is a typical microfluidic channel, which is often used to study microbubble generation, growth, and manipulation in a confined space [10,11,12,13,14]. Recently, microbubbles can be manipulated inside a blood vessel [15]. Due to the symmetry of the circular cross-section of a capillary tube, the tube is always used as a model to investigate the flows in a confined space theoretically and numerically [16]. Experimental studies of bubble growth inside a tube may help to verify the theoretical and numerical results of flows, which will help to manipulate microbubbles inside blood vessels precisely.

First, the manipulation of microbubbles in a capillary tube in a microfluidic channel requires the generation of microbubbles. In recent years, a common and convenient method to generate microbubbles is induced by laser heating. Laser-induced microbubbles are generally produced by the thermal effect of radiation with either a continuous-wave (CW) laser [17,18,19,20,21,22,23,24,25] or a pulsed laser [26]. Materials are used for heating to create microbubbles include absorbing substrates [27,28,29,30,31], absorbing liquids [20,32], and light-absorbing particles (APs) [33,34,35]. Laser heating produces cavitation, which means that a superheated area (superheat limit for water is 270–302 °C) is created and there is explosive liquid evaporation at the focal point [20].

The process and mechanism of microbubble generation and growth are very complex in tiny spaces [36]. After the bubble is created, there is a Marangoni convection around the microbubble induced by laser heating and this can be indicated by microparticles [31]. For a bubble generated by laser heating of unfixed APs, the convective flow will collect APs at the laser heating point, and the AP aggregation affects the microbubble growth process. In most studies, the bubbles were generated by heating of absorbing substrates or position-fixed APs and the area of the laser heating does not change in the process. The growth process of laser-induced bubbles with increasing APs number is poorly investigated.

In this paper, we have studied the growth of microbubbles in capillaries where the heating area was changed by the Marangoni convection during the growth process. Micron-sized APs were used as both light-absorbing material and tracer particles. This allows the convection to be observed. The relationship between the growth of microbubbles and the number of APs gathered at the bottom of the microbubbles was investigated in capillary tubes. The effect of tube diameter on the velocity of convective flow and number of APs at the bottom of the bubbles is demonstrated by numerical simulation. At last, the forces on the APs at the bubble surface are discussed.

## 2. Materials and Methods

**Figure 1:**
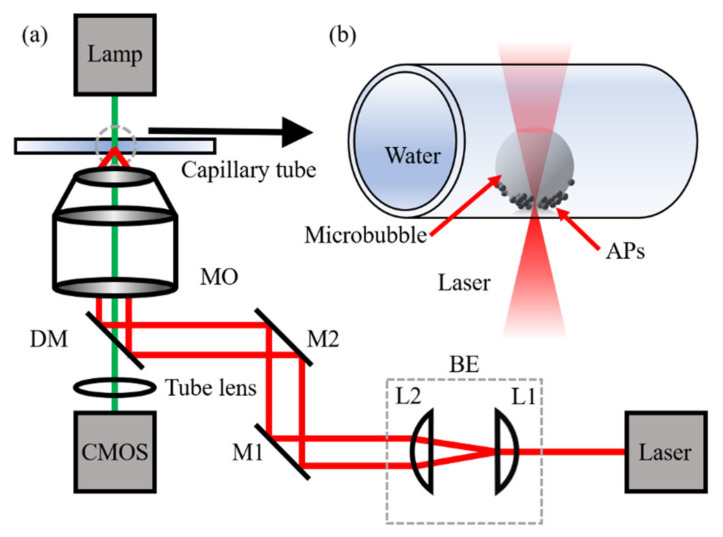
(**a**) Experimental setup. BE, beam expander; M1–M2, mirrors; DM, dichroic mirror; L1–L2, lenses; MO, microscope objective; CMOS, CMOS camera; (**b**) Schematic diagram of a microbubble in a capillary tube. The microbubble is generated by laser irradiation on APs. Some APs gather at the bubble bottom.

Our experimental setup is shown in Figure 1a, which is based on a previous paper by our group [37]. A 1064-nm laser (CNI, Changchun, China, MILN-1064, TEM_00_, CW) with a beam waist of 3 mm is used to create a heat source. The laser beam is expanded to fulfill the pupil of the objective (6 mm) with the beam expander, which consists of two lenses (L1 and L2, *f_L1_* = 30 mm, *f_L2_* = 100 mm). After being reflected by two mirrors, the laser beam is reflected upwards by a dichroic mirror to the microscope objective and focused on the inner bottom of a capillary tube. To investigate the effect of curved walls on the generation of microbubbles, we used four glass capillary tubes with different inside diameters (*d* = 0.2 mm, 0.3 mm, 0.5 mm, 0.8 mm) to contain the sample. The capillary tube is fixed on a three-axis stage. Images are captured by a CMOS camera. The objective we used in experiment is a 40× objective, the numerical aperture is 0.7, and the working distance is 3.76 mm.

The sample is a diluted suspension of APs which are core-shell magnetic silica microspheres (Affimag SLE, Fe_3_O_4_@SiO_2_, 4.0–5.0 μm in diameter, BaseLine Company, Tianjin, China). The shell material (Fe_3_O_4_) is a good light-absorbing material. The AP diameter is measured with microscopic images and is 4.51 ± 0.27 μm. The initial concentration (*c*) is about 6 × 10^7^ cm^−3^ in the suspension and the suspension solvent is phosphate buffer saline. The APs were diluted in deionized water for the experiments. The generated microbubble is confined at the bottom of a capillary tube when the beam is focused on the APs, as shown in Figure 1b. All experiments were performed at room temperature.

## 3. Results

We use a laser to irradiate the APs to generate microbubbles in circular capillary tubes and study the factors influencing the growth process of microbubbles by parameters such as laser power, capillary wall curvature, and number of APs.

### 3.1. Microbubble Generation in Capillary Tube

**Figure 2:**
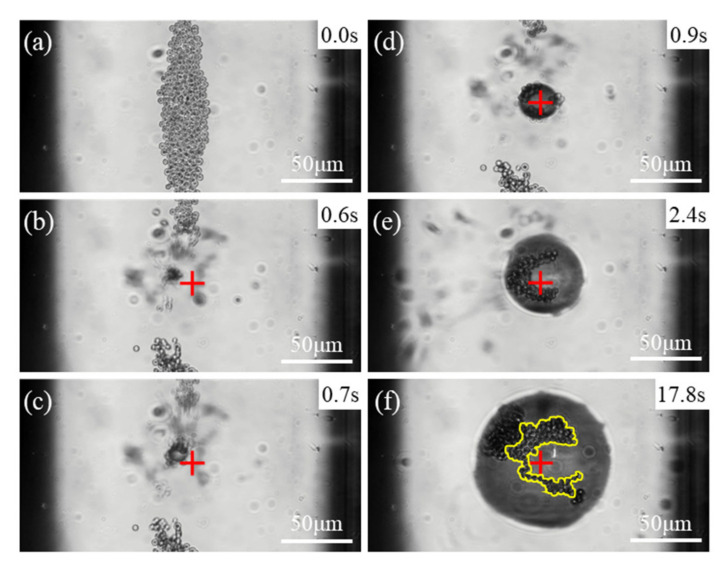
Microbubble generation and growth in a capillary tube with diameter of 0.2 mm. *c* = 1.2 × 10^7^ cm^−3^. *P* = 189 mW. “+” indicates the laser focal spot. (**a**) The laser was off and the APs settled at the bottom of the capillary tube. (**b**–**e**) Laser was on and a microbubble was generated and grew; some APs gathered at the bottom of it. (**f**) The yellow outline marks the APs at the bottom of the microbubble when bubble size is stable and they are stationary.

After injecting sample into a capillary tube, the APs settled to the bottom of the capillary tube. When the laser irradiated the APs, a microbubble generated and grew to a stable size. Figure 2 shows screenshots of the experiment inside a 0.2 mm capillary tube. In Figure 2a, the laser was off at first and the APs settled at the bottom of the capillary tube. In Figure 2b, when the laser irradiation began, it can be seen that a small microbubble has generated. In Figure 2c–f, the microbubble was captured at the focal spot of the laser beam and continued to grow under continuous laser heating. In Figure 2e, it can be seen that more APs were brought into the microbubble bottom by convection. Due to obstruction of the microbubbles, they can no longer leave the bottom in most circumstances. Finally, the size of the microbubble stabilized and the diameter at this state was defined as the stable diameter (*D_S_*). Almost no free APs can be seen in the field of view in Figure 2f when the microbubble has grown to its stable size.

In Figure 2f, the APs at the bottom of the microbubble are stable at the focal plane and marked by a yellow outline for calculating the area. Unlike other APs attached to the surface of the microbubble, which can move, these APs are stationary at the bubble bottom. These APs absorb the laser energy which is essential for the microbubble growth. The area of these APs is used as an indicator equivalent to the number of them.

The growth of bubbles is a complex process. The current studies show that bubble formation is caused by the evaporation of water that reaches boiling point upon laser illumination. Additionally, the bubble growth is dominated by the diffusion of dissolved gases after the microbubble is created [22,38,39].

### 3.2. Effect of Laser Power on Microbubble Growth

**Figure 3:**
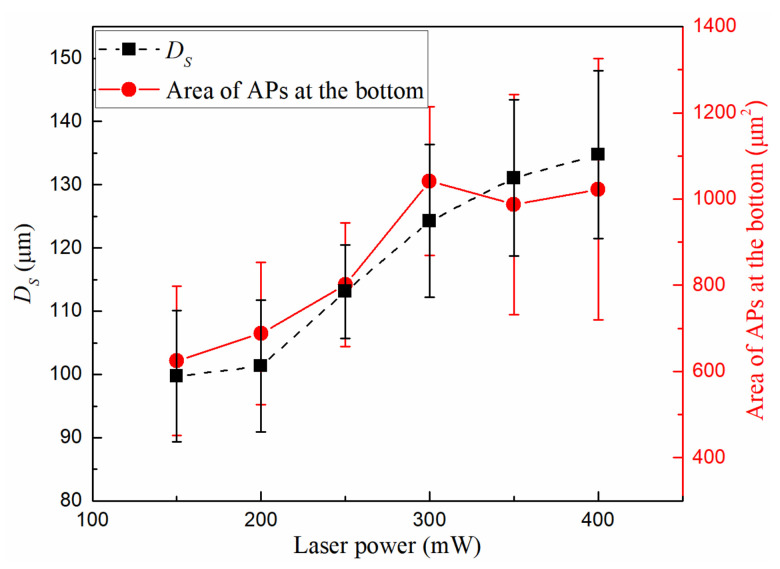
*D_S_* at different laser power. The area of the APs at the microbubble bottom was drawn with a red solid line. Left vertical coordinate is *D_S_* and right vertical coordinate is the area of the APs. *c* = 3.0 × 10^6^ cm^−3^.

We used lasers with different powers to generate microbubbles in a 0.3 mm tube with the same sample concentration (20 times diluted) and measured the *D_S_* of 10 microbubbles at each power. The areas of the APs were calculated for each power. The results are shown in Figure 3. When the laser power increases from 150 mW to 400 mW, the stable bubble diameter increases from 99.7 μm to 134.7 μm and the area of the APs increases from 625 μm^2^ to 1025 μm^2^. From these trends, it can be seen that increasing the laser power causes more APs to gather and be confined at the microbubbles bottom, meanwhile, the microbubbles are also getting larger. These can be explained as the effect of laser power on convection range. The higher the temperature at the laser focal spot, the larger the convection range, and the more APs will enter into the microbubble bottom.

### 3.3. Effect of Wall Curvature on Microbubble Growth

**Figure 4:**
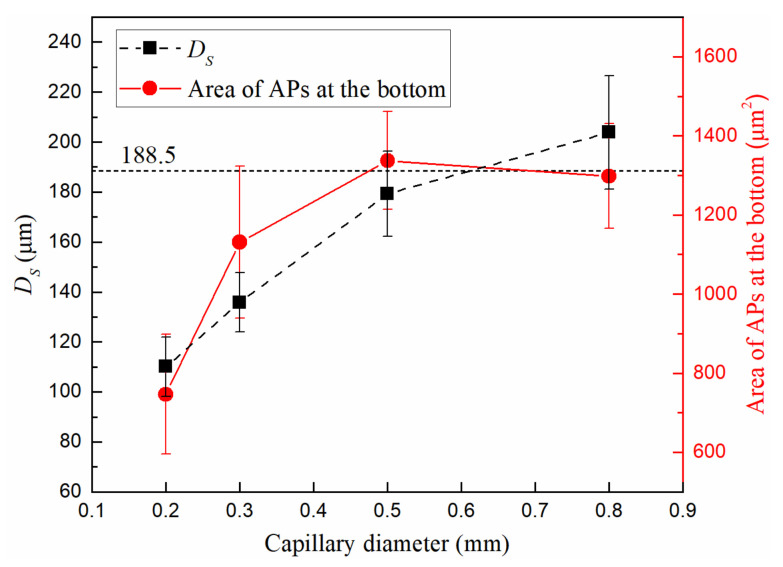
*D_S_* in different diameter (*d* = 0.2 mm, 0.3 mm, 0.5 mm, 0.8 mm) capillary tubes. The horizontal dashed line is the diameter of the microbubbles produced on the slide under the same experimental conditions. The area of APs at the microbubble bottom was drawn with a red solid line. *c* = 3.0 × 10^6^ cm^−3^. *P* = 189 mW.

We used four different diameter (*d* = 0.2 mm, 0.3 mm, 0.5 mm, 0.8 mm) capillary tubes to investigate the effect of curved walls on the generation and growth of microbubbles. We diluted the sample 20 times, set the laser power to 189 mW, and measured the *D_S_* of 10 microbubbles in each capillary tube. The results are shown in Figure 4. At the same concentration and laser power, when the tube diameter increases from 0.2 μm to 0.8 μm, the stable bubble diameter increases from 110.0 μm to 203.9 μm and the area of the APs increases from 747 μm^2^ to 1298 μm^2^.

In order to compare with the microbubbles without the influence of a curved wall, we measured the average microbubble diameter of 188.5 μm on a glass slide under the same experimental conditions. It can be seen that there is very little difference in the size of the microbubbles generated in the capillary tube with diameters of 0.8 mm and on the glass slide. It can be concluded that in a capillary tube with a diameter of more than 0.8 mm, the tube wall has almost no restriction on the growth of microbubbles.

There may be two aspects which count for the trends in Figure 4. One is the restriction of the Marangoni convection by the curved wall, i.e., the Marangoni convection collects APs within a certain range to the microbubble bottom or other locations on the surface and wall curvature may have an effect on the velocity and range of convection, which will be discussed in Section 4.1. The other is the influence of the curved wall on the number of APs in the convection range. When samples with same concentration were injected into two capillary tubes with different diameters, the number of APs settled in the larger diameter capillary tube is significantly more than that in the smaller one. This causes more APs stabilizing at the bottom of the bubble in larger diameter tubes. More APs may absorb more energy of the laser beam, which may affect the stable bubble size. To eliminate this influence, we conducted experiments to investigate the relationship between microbubble size and AP area by adjusting the concentration of the suspension so that the number of APs stabilizing at the bottom of the bubble is within a certain range.

### 3.4. D_S_ Changed with Area of APs at Microbubble Bottom

**Figure 5:**
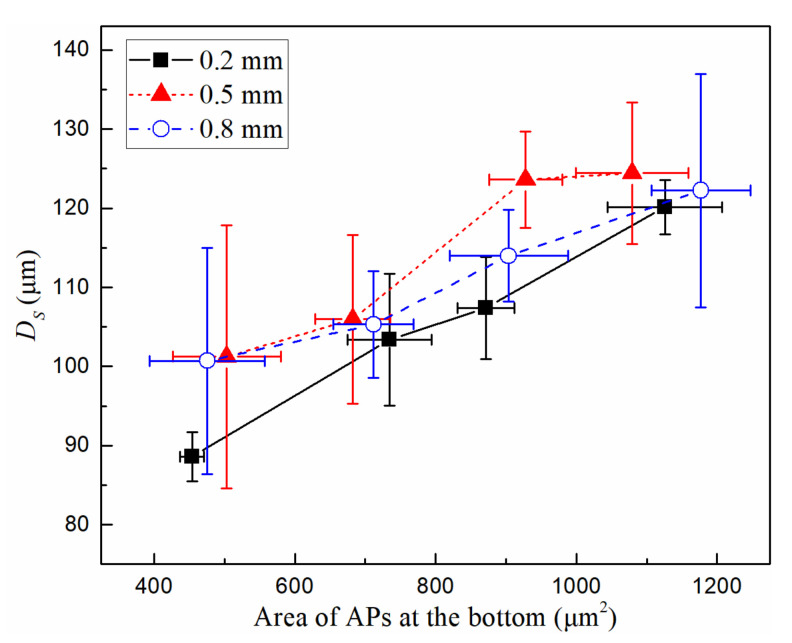
The relationship between *D_S_* and the area of APs at the bottom in the tubes with different diameters. Starting from 400 μm^2^, a group of data in the range of every 200 μm^2^ was obtained for this graph. *P* = 189 mW.

From Figure 3 and Figure 4, it can be seen that the size change of the microbubble is often accompanied by change of the area of the APs at the bottom. To measure the direct effect of the area of the APs at the bottom on microbubble size, we adjusted the sample dilution factor so that the AP area at the bottom in different tube diameters was in the same range (approximately 400–1300 μm^2^). The relationship between *D_S_* and the area of APs at the bottom was tested in three capillary tube diameters with the same power of 189 mW. The results are shown in Figure 5. The bubble size increases with the increasing area of APs at the bubble bottom. These APs absorb the laser energy which determines the final bubble size at same laser power.

Under the same heating conditions, as the specific heat capacity of Fe_3_O_4_ (619 J·kg^−1^·K^−1^) is much smaller than that of water (4182 J·kg^−1^·K^−1^) [40], the more APs in the heated area, the easier the temperature will rise and stronger convection will be generated, while more air will precipitate from the suspension for bubble growth.

In most cases, the APs are at the bottom of the microbubbles surrounding the laser spot. In a few cases, the APs gathered directly in the center of the microbubble bottom, i.e., the APs were irradiated directly by the central spot and the microbubbles grew rapidly and unstably, causing them to float or break up in a short time. This case is not counted in the data here.

The radius of the laser focal spot is about 10 μm, while APs are about 10 μm from the laser center. We can speculate that during the bubble growth, although the APs are not in the center, APs near the center can still be heated by the edge of laser beam and conduct heat to surrounding APs to form a hot area and induce a temperature gradient.

## 4. Discussion

### 4.1. Convection in Capillary Tubes

**Figure 6:**
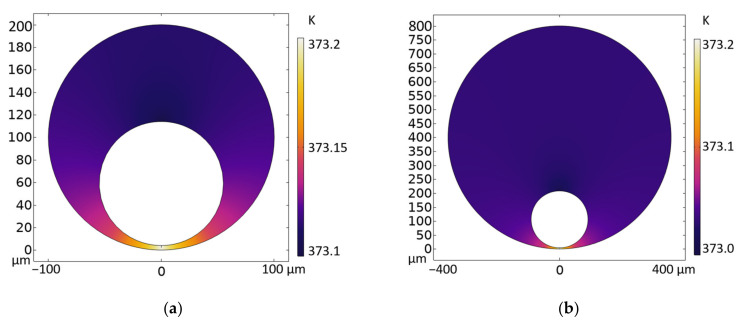
Simulated 2D temperature distribution maps for microbubbles generated in capillary tubes. (**a**) 0.2 mm diameter capillary tube; (**b**) 0.8 mm diameter capillary tube.

The area of the APs at the bottom is formed by convection, which is affected by laser power and tube wall curvature. The convection velocity increases with the increasing laser power. In order to study the influence of tube wall curvature on convection, we used COMSOL Multiphysics, a finite-element mode solver, to simulate Marangoni convection in 0.2 mm diameter and 0.8 mm diameter capillary tubes. The following equation describes the forces generated by the Marangoni effect at the interface (liquid/gas) [41]:

(1) $$\eta \frac{\partial u_{x}}{\partial y}=\gamma_{T}\frac{\partial T}{\partial x},$$

where *η* is the dynamic viscosity coefficient of the liquid, which was set as 1 mPa·s in this simulation, *u_x_* is the tangential component of the fluid velocity vector at the interface (liquid/gas), *x* and *y* are the unit vectors tangent and normal to the interface, respectively, *T* is the temperature, and *γ_T_* is the temperature derivative of the surface tension (N/(m·K)). Equation (1) indicates that the shear stress on the surface is proportional to the temperature gradient. We simplified the capillary tube into a two-dimensional model and set the microbubble size as the average data measured by the experiments. The heating point temperature was set as 373.2 K. We considered the different sizes of the microbubbles in 0.2 mm and 0.8 mm diameter capillary tubes. If the temperature gradient is assumed to be the same, the temperature at the top of the microbubbles was set as 373.1 K and 373.0 K, respectively. The temperature distribution maps are shown in Figure 6.

**Figure 7:**
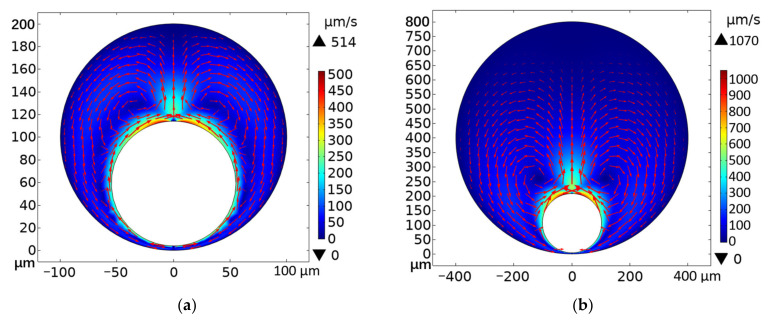
Simulated 2D convective velocity maps for microbubbles generated in capillary tubes. The arrow indicates the direction of the flow field and the color indicates the velocity magnitude. (**a**) 0.2 mm diameter capillary tube; (**b**) 0.8 mm diameter capillary tube.

The velocity simulation results are shown in Figure 7, from which we can see that the maximum convection velocity in the 0.8 mm capillary tube reaches 1070 μm/s, while the maximum convection velocity in the 0.2 mm tube is 514 μm/s. The simulation results are in the same order of magnitude as the results in [42]. The reason for the different convection velocity in the two diameters of capillary tubes is the difference in space confinement. In other words, the smaller diameter of the tube wall limits convection more significantly. The results indicate that because of the different convection velocity, the effective area of the convective forces, i.e., the area in which free APs can be pushed and gathered towards microbubbles, is larger in the 0.8 mm diameter capillary tube (1298 μm^2^ in experiment) compared to the 0.2 mm diameter capillary tube (747 μm^2^ in experiment). Therefore, when the other experimental conditions are the same, the microbubbles generated in the larger diameter capillary tube are larger than those generated in the smaller diameter capillary tube.

We believe that the convection velocity determines the effective area of the convection (i.e., the area where APs can be pushed towards the bubble) and affects the number of APs that the bubble can collect. However, due to equipment limitations (mainly the frame rate of the CMOS camera), the critical velocity for pushing APs is difficult to measure. So, based on the simulation, we can only give semi-quantitative results for the time being, as well as use the maximum velocity in the simulation to explain the reason why the APs are stable at the bottom rather than moving with convection, in the following part.

### 4.2. Analysis of Forces on an AP at Bubble Surface

**Figure 8:**
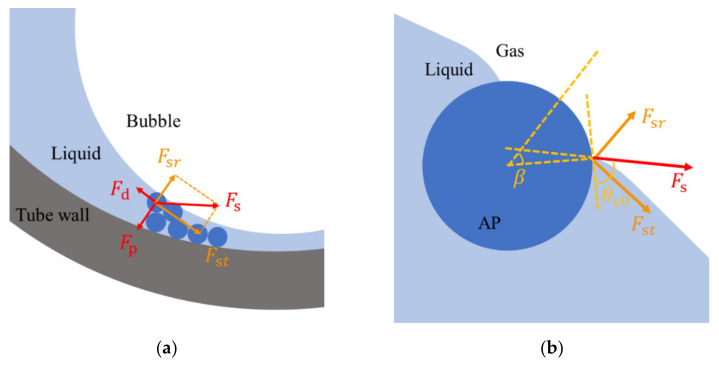
Schematic figure of the forces on an AP at the bubble surface. (**a**) The pressure force and the surface tension force balance in the radial direction; (**b**) Diagram of the surface tension force.

When APs gathered at the bottom of the microbubble, there was stacking in the outermost APs observed in the experiments. The upper APs are confined by the bubble, allowing the bottom APs to be stabilized. To explain why the APs are still rather motive, we analyze the forces on an AP at the bubble surface. An AP at the upper layer is mainly subjected to the following forces: surface tension force *F_s_*, pressure force *F_p_*, and convective drag force *F_d_*, as described in Figure 8a. The *F_s_* can be calculated by the following equation [29]:

(2) $$F_{s}=\gamma \cdot 2\pi R\cdot \sin\beta ,$$

where *γ* is the surface tension (~70 mN/m for water at room temperature [40]), *β* is the half central angle, and *R* is the radius of the AP, as shown in Figure 8b. The radial component of *F_s_* is as follows:

(3) $$F_{sr}=F_{s}\sin(\left|\beta -\theta_{c0}\right|),$$

which can be balanced by the pressure force [29] as follows:

(4) $$F_{p}=\pi {\left(R\sin\beta \right)}^{2}\frac{2\gamma }{R_{B}},$$

where *θ_c_*_0_ is the contact angle between the AP and the liquid–gas interface and *R_B_* is the radius of the bubble. The two forces can be compared as follows:

(5) $$\frac{F_{sr}}{F_{p}}=\frac{R_{B}}{R}\frac{\sin(\left|\theta_{c0}-\beta \right|)}{\sin\beta }.$$

In the experiment, *R_B_* and *R* were estimated to 50~100 μm and 2.25 μm, respectively, which means that sin*β* is at least 20 times larger than sin(|*θ_c_*_0_*−β|*). Therefore, *β* ≈ *θ_c_*_0_. The tangential component of *F_s_* is as follows:

(6) $$F_{st}=F_{s}\cos(\left|\theta_{c0}-\beta \right|)\approx F_{s}\approx \gamma \cdot 2\pi R\cdot \sin\theta_{c0}.$$

For Fe_3_O_4_, the typical value of *θ_c_*_0_ is 85.36° [43] and *F_st_* is ~1 mN. *F_d_* can be calculated using Stokes’ law as follows:

(7) $$F_{d}=6\pi R\eta v.$$

As shown in Figure 7, the upper limit of *v* of the Marangoni convection is less than 1000 μm/s, so the upper limit of *F_d_* is ~42 pN, which is much smaller than *F_st_*. The buoyancy is 0.7 pN and gravity is 1.7 pN. The thermophoresis force (~1 pN) is one order of magnitude smaller than the drag force for the outermost APs. The calculations show that the surface tension force is dominant for the APs at the upper layer. Those APs are fixed at the bubble surface, which makes the APs at the lower layer embedded in the void therein. Therefore, the APs at the bottom of the microbubble are still rather motive under the convection.

In recent years, a laser was used to generate convections and corresponding drag forces in solutions [44,45,46]. We think drag force can be used to measure cell deformability with higher efficiency relative to optical tweezers. This is because the convection can apply drag force on multiple cells at the same time, whereas optical tweezers can only deal with cells one by one. Here, Marangoni convection forms when a microbubble generates. We think that the convective drag force can be used to aggregate cells and measure the deformability of cells with high efficiency.

## 5. Conclusions

In this study, we used a laser to irradiate APs to generate microbubbles in capillary tubes. The microbubbles are confined at the bottom of the capillary tubes due to thermocapillary force. Firstly, the process of microbubbles generation and growth in a capillary tube was demonstrated. Experiments have shown that microbubbles can be generated in capillary tubes of different diameters. Secondly, factors affecting the growth of the microbubbles were investigated. The experimental results show that laser power, wall curvature, and number of APs at the bottom of the microbubbles all have an effect during microbubble growth. The microbubble size increases with increasing laser power when the same concentration of APs is used in the same capillary tube. At the same laser power and sample concentration, the microbubble size becomes larger as the diameter of the tube becomes larger. We conclude that the number of APs gathered at the bottom of the microbubbles is a key factor affecting the size of the microbubbles. While in the same concentration of AP samples, laser power and capillary wall curvature exert effects on the number of APs gathered at the bottom of the microbubbles. Finally, we investigated the effect of wall curvature on the number of APs gathered on the bottom of the microbubbles by numerical simulation, which indicates that the curvature of the capillary tube wall will affect the speed of the Marangoni convection. The larger the diameter of the capillary tube, the larger the convection range, and the more APs will be collected at the bottom of the microbubbles. Calculations of the forces on an AP show that the surface tension force is much greater than the convective drag force on the APs in direct contact with the microbubbles, so the APs are stationary under the convection.

Our work provides experimental observations of position information of APs during growth of laser-induced microbubbles in capillary tubes and investigates the key parameters affecting the growth of laser-induced microbubbles, providing a reference for subsequent studies on the dynamical mechanism of microbubble growth.

## Data Availability

Not applicable.
